# Investigation of the structural, electronic, and optical properties of Mn-doped CsPbCl_3_: theory and experiment

**DOI:** 10.1039/c9ra05685h

**Published:** 2019-09-18

**Authors:** Nivedita Pandey, Abhishek Kumar, Subhananda Chakrabarti

**Affiliations:** Department of Electrical Engineering, Indian Institute of Technology Bombay India-400076 nivedita@ee.iitb.ac.in princeabhi@ee.iitb.ac.in subho@ee.iitb.ac.in

## Abstract

Wide energy gap inorganic halide perovskites have become emerging candidates for potential applications in modern optoelectronics devices. However, to date, these semiconducting compounds have not been explored theoretically to a significant extent. Herein, we performed *ab initio* computations to explain the structural, electronic and optical behaviour of inorganic CsPbCl_3_ and Mn-doped CsPbCl_3_ nanocrystals (NCs). We also synthesized these NCs and further validated our experimental results with density functional theory (DFT) calculations. The results provide insight into the effect of Mn doping on the important properties of CsPbCl_3_ NCs such as their lattice parameter, electronic band structure, density of states, dielectric constant, absorption coefficient and refractive index. After geometry optimization using the Limited-memory Broyden–Fletcher–Goldfarb–Shanno (LBFGS) algorithm, a reduction in the lattice parameter from 5.605 Å to 5.574 Å was observed after doping Mn in the CsPbCl_3_ NCs, which is in good agreement with the calculated results from the X-ray diffraction (XRD) pattern (5.610 Å to 5.580 Å) and high-resolution transmission electron microscopy (HRTEM) images (5.603 Å to 5.575 Å). The incorporation of Mn in CsPbCl_3_ was observed in the electronic band structure in the form of additional states present in the energy gap and an increment in the band gap of the CsPbCl_3_ NCs. This result is consistent with the photoluminescence (PL) plot, which showed dual color emission in the case of the Mn-doped CsPbCl_3_, which is attributed to the Mn^2+^ d-band to d-band transition. The partial density of states (PDOS) of the Mn-doped CsPbCl_3_ NCs clearly indicates the contribution of the Mn 3d orbitals to the upper valence band and conduction band together with the contribution of the Pb 6p and Cl 3p orbitals. Moreover, a blue-shift phenomenon was observed from the dielectric constant and absorption coefficient spectra, which is due to the incorporation of Mn in CsPbCl_3_. Also, a significant peak was observed in the absorption coefficient and dielectric constant spectra around 2.08 eV, which is in good agreement with the PL plot. This DFT study with experimental observation provides a way to investigate this type of compound and to tailor its interesting characteristics through doping.

## Abstract 2

Wide energy gap inorganic halide perovskites have become emerging candidates for potential applications in modern optoelectronics devices.

## Graphical abstract

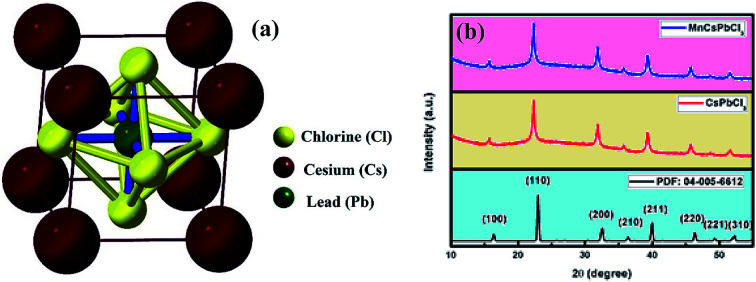

## 1. Introduction

Solar energy plays a significant role in the quest for green and renewable sources of energy worldwide. Solar energy has become a good substitute for the different sources of energy, which can overcome the drawbacks of the traditional sources of energy.^1^ To utilize this energy, solar cells play a significant role in transforming light energy into electrical energy with minimal loss together with a low emission of greenhouse gases.^2^ In the design of modern electronic devices such as light-emitting diodes (LEDs)^3^ and photovoltaic cells,^4–7^ inorganic perovskites have attracted significant attention due to their low cost and promising utility. Thus, in the solar cell research community, inorganic perovskites^8–14^ have attracted significant interest in recent years. The general structural formula for these compounds is ABX_3_, where A represents the inorganic cation, B the divalent metallic cation and X the halogen. Inorganic perovskites show unique and interesting properties, such as significant absorption coefficient and good semiconducting behavior, which make them suitable candidates for widespread utility in photovoltaic and optoelectronic devices.^15–20^

Cesium lead halide perovskite (CsPbX_3,_ X= Cl, Br and I),^21^ which is an inorganic perovskite, has attracted growing attention due to its fascinating properties of extremely efficient PL, which can be tailored over the whole visible spectrum by controlling dimension and anion in its NCs. It has been used as an active material in a wide applications such as light-emitting diodes (LEDs),^22^ photovoltaic cells^23^ and lasers.^24^ Particularly, perovskites can be used as promising materials in photovoltaic applications, in which, within seven years the efficiency has been upgraded from 3.8%^25^ to 22.1%.^26^ Thus, this perovskite has been developed as a potential material in the light-emitting field. Light-emitting modern devices in the visible range can be tuned by controlling the NC size. However, hybrid organic–inorganic lead halides have a major limitation of sensitivity towards light, heat, and humidity. Accordingly, the CsPbX_3_ inorganic perovskite has been proven to be superior to other hybrid organic–inorganic lead halide perovskites due to its ability to show optical properties^27^ with higher stability.^28^ Also, CsPbX_3_ nanocrystals are much more superior than other NCs due to their tunability, leading to their wide application in lasers based on single^27^ and multiphoton pumping,^29^ LEDs^30^ and in optoelectronics.

Nowadays, there are many advantages and applications of cesium lead halide perovskites. However, the highly toxic nature of lead is a serious concern, which has a hazardous impact on the environment and health of human beings on a commercial level.^31^ Accordingly, the synthesis of lead-free perovskites is urgent to completely eliminate lead, which causes serious health and environmental issues. Additionally, the optical, electronic and magnetic characteristics of NCs can be controlled by incorporating impurity ions. Recently, there have been notable efforts on doping aimed at the complete elimination/reduction of lead. Doping of impurities has been done in II–VI and III–V NCs to introduce magnetism,^32^ impurity-based PL^33^ and induction of p- and n-type behavior.^34^ The impurity dopants that are widely studied and effectively impart novel properties in semiconductor nanocrystals are Mn^2+^,^35^ Co^2+^,^36^ Cu^2+^,^37^ and Ag^+^.^38^ Among them, Mn^2+^ doping has been widely studied^35,39-42^ due to its high abundance, and potential to induce optical and magnetic properties^43^ in the doped host semiconductor nanocrystals.

Herein, we examined the effect of Mn as a dopant in CsPbCl_3_ on its structural, electronic, and optical behavior. To obtain information about the crystal structure, XRD was performed and the results compared with the JCPDS database. Further, to study the morphology of the synthesized samples, TEM and high-resolution transmission electron microscopy (HRTEM) were performed. Moreover, optical and electronic characteristics were studied using PL and ultraviolet-visible (UV-vis) spectroscopy. A change in the energy band gap was observed in the case of Mn-doped CsPbCl_3_. These experimentally observed results were compared with the computed results obtained from the DFT-based *ab initio* study, providing deep insight into the unique and interesting electronic and optical properties of the samples. The DFT-based theoretically computed results show very good agreement with our experimentally observed results.

## 2. Computational models and methods

Herein, we explored the structural, electronic and optical properties of CsPbCl_3_ and Mn-doped CsPbCl_3_ compounds applying *ab initio* computations based on density functional theory (DFT). The computations were carried out using the Quantumwise Atomistix (ATK) software package.^44^ Here, *ab initio* computations were performed using the generalized gradient approximation (GGA) with the Perdew–Burke–Ernzerhof (PBE)^45^ exchange-correlation functional. The Brillouin zone was sampled using the Monkhorst-Pack grid^46^ with 9 × 9 × 9 sampling points. To achieve high accuracy in our calculations, an energy cut-off of 75 hartree was used. The crystal structure of the compounds was optimized using the LBFGS optimization code until the residual force on each atom was less than 0.05 eV Å^−1^. A norm-conserving FHI (Fritz Haber Institute) pseudopotential was used for our calculations. In this work, the double zeta polarized (DZP) basis set was chosen for all atoms, which is comprised of three basis orbitals (analytical split, confined orbital, and polarization orbital for the first unfilled shell of an atom). In our calculation, the Mn atom was doped in CsPbCl_3_ at the Pb site and simulations were performed on a 2 × 2 × 2 supercell, as shown in Fig. 2(a) and (b).

To investigate the optical behavior of CsPbCl_3_ and Mn-doped CsPbCl_3_, the Kubo–Greenwood formalism was used in the DFT framework. The meta-generalized gradient approximation (MGGA) with Tran and Blaha (TB09)^47^ as the exchange-correlation functional was utilized to compute various optical properties, such as dielectric constant, absorption coefficient, and refractive index. A *k*-point sampling of 15 × 15 × 15 was chosen to compute the optical behavior mentioned above for CsPbCl_3_ and Mn-doped CsPbCl_3_. We had accounted for the thermal effect while calculating the optical properties by choosing a broadening of 0.01 eV in the simulations.

The susceptibility tensor is given by the Kubo–Greenwood formalism as:i
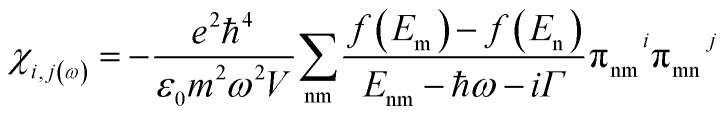
where, π_nm_^*i*^ represents the *i*^th^ component of the dipole matrix element between two states n and m, *V* represents the volume, *Γ* represents the broadening and *f* represents the Fermi function.

From the above eqn (i), the relative dielectric constant *ε*_r_ is related to the susceptibility according to Griffiths^48^

ii *ε*_r_ = (1 + *χ*(*ω*))

The refractive index, *η*, is related to the extinction coefficient, *κ*, and the relative dielectric constant, *ε*_r_, asiii
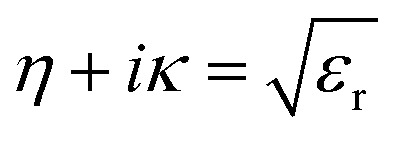


Finally, the optical absorption coefficient is given by *α* as in ref. 49.iv
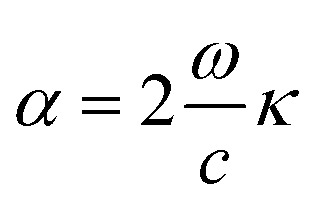


## 3. Experimental

### 3.1 Chemicals

The chemicals utilized in our synthesis were obtained from Sigma Aldrich and used as received. Cesium carbonate (Cs_2_CO_3_, 99%), lead chloride (PbCl_2_, 99%), manganese chloride (MnCl_2_·4H_2_O, 99%), 1-octadecene (ODE, ≥90%), oleic acid (OA, 90%), oleylamine (OAm, 90%), acetone (≥99.9%), and hexane (≥95%) were used in our synthetic procedure.

### 3.2 Synthesis

#### 3.2.1 Preparation of Cs-oleate solution

Following the synthetic method reported in ref. 21, in a 250 mL 2-neck flask, 0.325 g of Cs_2_CO_3_ was dissolved in 2 mL of OA and 20 mL of ODE. This mixture was heated at a temperature of 110 °C for 60 minutes under a nitrogen (N_2_) atmosphere. Further, it was heated at 140 °C for 30 min. Subsequently, the obtained oleate of Cs was heated at 110 °C for the preparation of CsPbCl_3_ and Mn-doped CsPbCl_3_.

#### 3.2.2 Preparation of CsPbCl_3_ and Mn-doped CsPbCl_3_

The synthesis of CsPbCl_3_ and Mn-doped CsPbCl_3_ was done using the previously reported method in ref. 50. Briefly, one mole of PbCl_2_ with 2.4 mL of OAm, 2.4 mL of OA, and 10 mL of ODE were added to a 150 mL 3-neck flask and heated at a temperature of 105 °C for 30 min under a nitrogen atmosphere. Afterwards, the temperature was increased to 160 °C and the precursors in the solvent were heated for 20 min. Subsequently, to solubilize the salt, the mixture was heated at an increased temperature of 200 °C. 1.6 mL of Cs-oleate solution was introduced by decreasing the temperature from 200 °C to 175 °C. An ice-water bath was used to immediately quench reaction mixture to room temperature. Manganese (Mn) was introduced as a dopant in CsPbCl_3_ by adding equal mol of MnCl_2_·4H_2_O to the above precursor. To synthesize the Mn-doped CsPbCl_3_ (Mn-doped CsPbCl_3_), all other steps were the same as above. The nanocrystals (NCs) were isolated by centrifugation at 10 000 rpm for 10 min. Further cleaning was performed using acetone, and the NCs were redispersed in hexane for further characterization.

## 4. Results and discussion

**Fig. 1:**
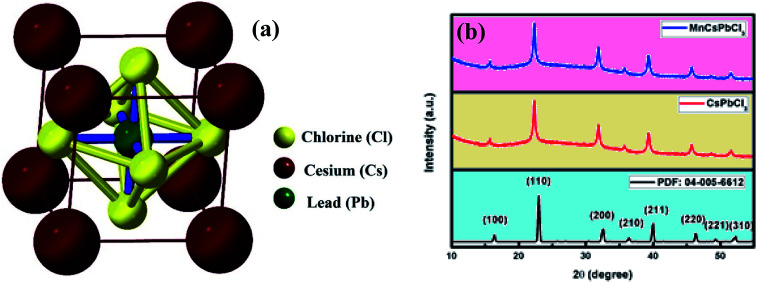
(a) Typical unit cell of the CsPbCl_3_ inorganic perovskite, where Cs^+^, Pb^2+^, and Cl^−^ occupy the corner, body centered, and face centered positions, respectively. (b) X-ray diffraction pattern of CsPbCl_3_ and Mn-doped CsPbCl_3_ nanocrystals (NCs).

CsPbCl_3_ and Mn-doped CsPbCl_3_ were synthesized using the hot-injection method, and the structural, optical, and electronic properties of the synthesized materials were also explored theoretically using DFT. Fig. 1(a) shows the unit cell of the CsPbCl_3_ inorganic lead halide perovskite, which consist of Cs (occupying corner position) and Pb (occupying body-centered position) as two cations with different sizes and Cl (occupying face-centered position) as the anion, which forms bonds with both Cs and Pb cations. The unit cell of CsPbCl_3_ consists of a PbCl_6_ cage (as shown in Fig. 1(a)), which plays a significant role in determining its electronic behavior.

In this work, we theoretically explored the effect of Mn doping in CsPbCl_3_ using the *ab initio*-based DFT simulation and further validated our calculated results with experimentally observed results. Fig. 1(b) shows the XRD patterns of both the undoped and doped CsPbCl_3_. The XRD patterns of these NCs were measured using an X'pert PANAlytical X-ray diffractometer with Cu Kα radiation (*λ* = 1.5406 Å) run a voltage of 40 kV and current of 30 mA. The observed XRD plots for our synthesized samples were compared with the JCPDS database (04-005-6612). The XRD pattern for CsPbCl_3_, as shown in Fig. 1(b), is consistent with the JCPDS database.

The effect of Mn doping can be clearly seen in Fig. 1(b) by analyzing the XRD pattern for the Mn-doped CsPbCl_3_. The XRD peaks for the Mn-doped CsPbCl_3_ shows the (110) peak at a higher angle compared to that for CsPbCl_3_. This shift towards a higher angle shows a reduction in the lattice parameter of the Mn-doped CsPbCl_3_ crystal structure.

**Fig. 2:**
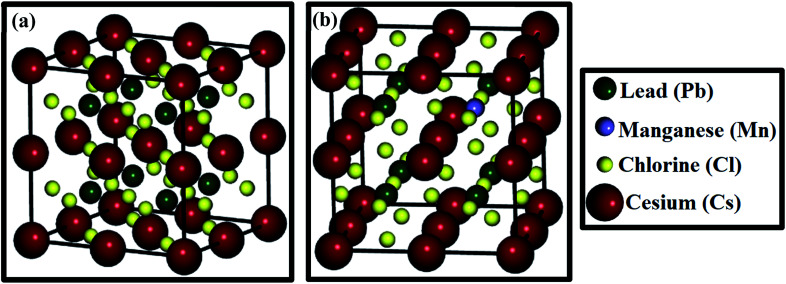
(a) Optimized crystal structure of 2 × 2 × 2 CsPbCl_3_ and (b) optimized crystal structure of 2 × 2 × 2 Mn-doped CsPbCl_3_.

The structural properties were explored using DFT calculations, in which geometry optimization of CsPbCl_3_ and Mn-doped CsPbCl_3_ was performed. Further, to study the ease with which Mn can be introduced within the CsPbCl_3_ lattice, the formation energy of Mn-doped CsPbCl_3_ was calculated by using the following equation:^51^

v *E*_f_ = *E*_Mn-doped_ − (*E*_undoped_ − *μ*_Pb_ + *μ*_Mn_)

where, *E*_undoped_ is the total energy of CsPbCl_3_ without Mn doping, *E*_Mn-doped_ is the total energy of Mn-doped CsPbCl_3_, *μ*_Pb_ is the energy of the Pb atom, and *μ*_Mn_ is the energy of the Mn atom. The energies of the Mn and Pb atoms were calculated from the total energies calculated for Mn molecules and Pb molecules, respectively. The calculated formation energy (*E*_f_) for Mn doped at the Pb site was found to be −4.3856 eV, which indicates the high stability of the Mn-doped CsPbCl_3_ crystal structure. Fig. 2(a and b) show the optimized 2 × 2 × 2 supercell of CsPbCl_3_ and Mn-doped CsPbCl_3_, respectively. After structure optimization, the CsPbCl_3_ crystal showed lattice parameters of *a* = *b* = *c* = 5.605 Å, which show good agreement with that in ref. 52, while the Mn-doped CsPbCl_3_ crystal structure showed lattice parameters of *a* = *b* = *c* = 5.574 Å. These results show a decrease in lattice parameters due to the introduction of Mn as a dopant in the CsPbCl_3_ crystal structure and confirm the cubic crystallinity of both CsPbCl_3_ and Mn-doped CsPbCl_3_, as reported in ref. 50. These calculated values are in good agreement with our experimentally obtained lattice parameters from the XRD patterns and HRTEM, as mentioned in Table 1.

**Table 1:** Comparison of the theoretically calculated lattice parameters from the *ab initio* calculations with the experimental results calculated using the XRD pattern and HRTEM

Compound	Theoretical (Å)	Experimental	
		XRD (Å)	HRTEM (Å)
CsPbCl_3_	5.605	5.610	5.603
Mn-doped CsPbCl_3_	5.574	5.580	5.575

**Fig. 3:**
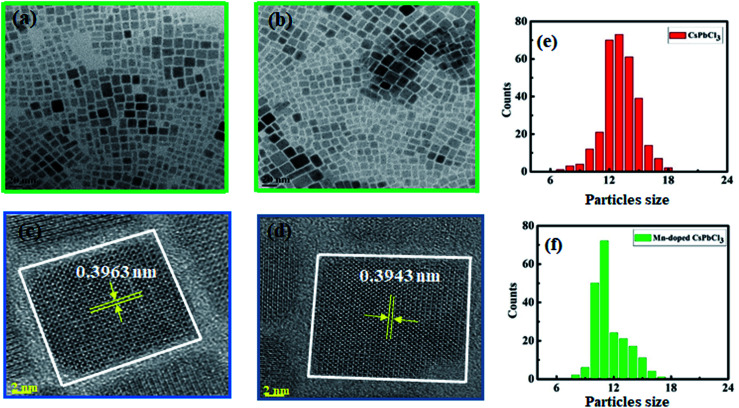
(a and b) TEM images, (c and d) HRTEM images and (e and f) calculated particle size of CsPbCl_3_ and Mn doped CsPbCl_3_, respectively.

TEM was performed to investigate the surface morphology and size of the synthesized compounds using a JEOL (JEM-2100F) field-emission gun transmission electron microscope (FEG-TEM). Fig. 3(a) and (b) show the TEM images of CsPbCl_3_ and Mn-doped CsPbCl_3_ NCs, respectively. In the TEM images, lattice fringes can be clearly seen all over the NCs, which indicate high crystallinity in both the CsPbCl_3_ and Mn-doped CsPbCl_3_ NCs. These results indicate the cubic crystal structure of the CsPbCl_3_ and Mn-doped CsPbCl_3_ NCs. Fig. 3(e) and (f) show the calculated size of the CsPbCl_3_ and Mn-doped CsPbCl_3_ NCs. Most of the NCs of CsPbCl_3_ have a size of 12.513 nm, while that for the Mn-doped CsPbCl_3_ is 10.591 nm. This clearly suggests that the reduction in the size of the NCs is due to the incorporation of Mn as a dopant in the CsPbCl_3_ NCs. To further study the atomic structure of the synthesized compounds, HRTEM was performed. The HRTEM images of the synthesized samples are shown in Fig. 3(c) and (d) for the CsPbCl_3_ and Mn-doped CsPbCl_3_ NCs, respectively. The observed interplanar spacing value from the HRTEM images for the (110) plane for the CsPbCl_3_ and Mn-doped CsPbCl_3_ NCs is 0.3963 nm and 0.3943 nm, respectively. Further, we calculated the lattice parameter values from the interplanar spacing values for the CsPbCl_3_ and Mn-doped CsPbCl_3_ NCs. The evaluated lattice parameter values are 5.603 Å and 5.575 Å for CsPbCl_3_ and Mn-doped CsPbCl_3_ NCs, respectively, which are consistent with that calculated from the XRD (110) peak and *ab initio* results. The observed reduction in the lattice parameter in the case of Mn-doped CsPbCl_3_ is attributed to the contraction in its lattice due to the smaller ionic radius of Mn^2+^ compared to that of the Pb^2+^ ion.

**Fig. 4:**
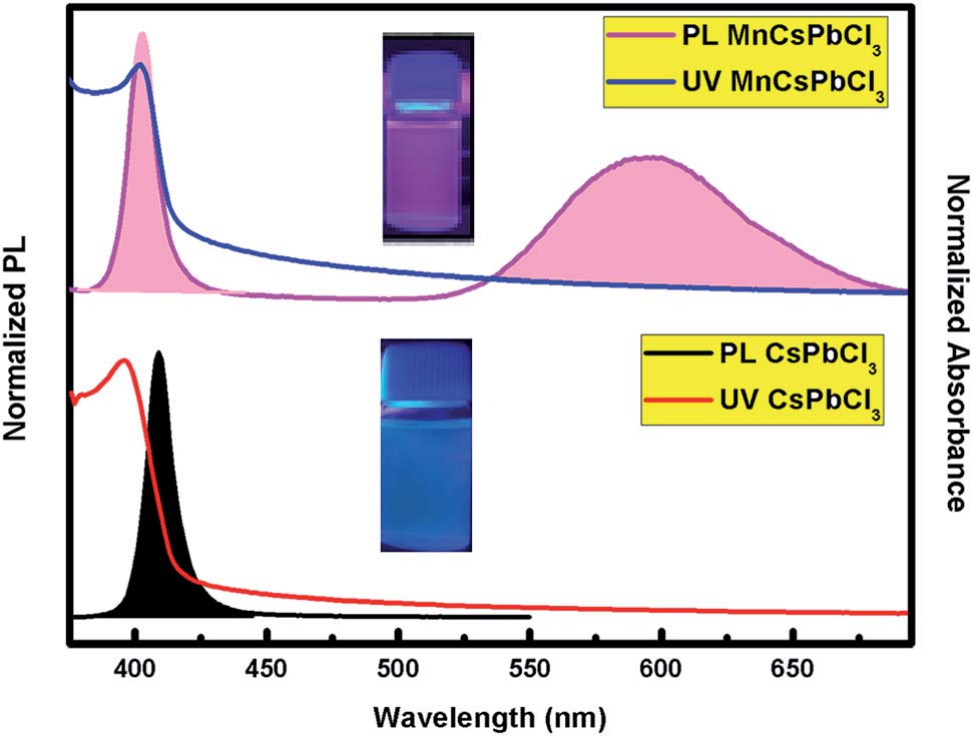
Photoluminescence (PL) and absorption spectra (UV-vis) of the undoped CsPbCl_3_ (bottom) and Mn-doped CsPbCl_3_ (top) NCs. The pictures in the inset show the compounds under UV excitation.

The optical behaviour of the CsPbCl_3_ and Mn-doped CsPbCl_3_ NCs were investigated by studying the photoluminescence (PL) and ultraviolet and visible absorption (UV-vis) spectra of their dispersion in hexane, as depicted in Fig. 4. The ultraviolet and visible absorption (UV-vis) spectra were measured using a PerkinElmer Lambda −950 UV-vis spectrophotometer. Photoluminescence (PL) spectra were collected using a HORIBA Scientific FluroMax 4 Spectrofluorometer. The absorption peaks were observed at around 416 nm and 398 nm for CsPbCl_3_ and Mn-doped CsPbCl_3_, respectively. The Mn-doped NCs showed a dual color broad peak emission, where one emission is located at 398 nm, while the other peak is at 584 nm, which is attributed to the Mn^2+^ d-band (^4^T_1_) to d-band (^6^A_1_) transition, as reported in ref. 50. The observed impurity peak in the Mn-doped CsPbCl_3_ is ascribed to the incorporation of the Mn^2+^ ion in the host CsPbCl_3_ NCs. The calculated values of full-width half maxima (FWHM) are 19 nm and 47 nm for the host CsPbCl_3_ and d-band to d-band transition of Mn^2+^ in Mn-doped CsPbCl_3_, respectively. These FWHM values for the synthesized NCs suggest the highly crystalline nature of these compounds. The inset pictures depict the luminescence of CsPbCl_3_ and Mn-doped CsPbCl_3_ solutions dispersed in hexane under 365 nm UV excitation.

**Fig. 5:**
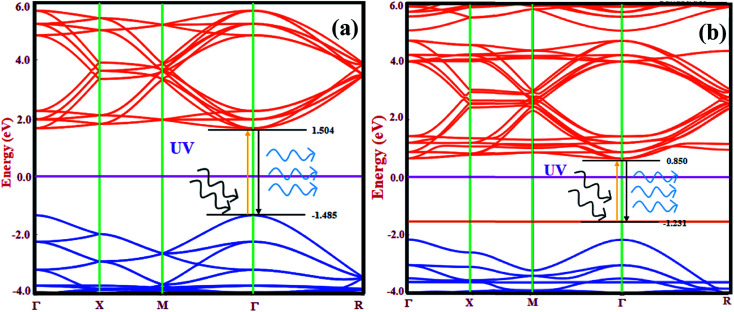
Electronic band structure of (a) CsPbCl_3_ and (b) Mn-doped CsPbCl_3_.

The electronic band structure contains significant information about the available energy for electrons, which determines the occupancy and non-occupancy of electrons in a particular energy state. The energy band gap in semiconductors and insulators are calculated by considering the separation between the valence band offset (VBO) and the conduction band offset (CBO). The electronic band structure also explains the transition of electrons from the VBO to CBO. Fig. 5(a) and (b) show the electronic band structure of CsPbCl_3_ and Mn-doped CsPbCl_3_, respectively along their highly symmetrized Brillouin zone (BZ) route.

By examining the band structure of CsPbCl_3_ in Fig. 5(a), the valence band offset lies at the center of the Brillouin zone (*Γ* point) with an energy −1.485 eV. The conduction band offset also lies at the *Γ* point with an energy 1.504 eV. This clearly indicates the direct band gap nature of the CsPbCl_3_ NCs with a band gap of around 2.989 eV. Thus, to investigate the effect of Mn as a dopant on the electronic properties of CsPbCl_3_, the electronic band structure of Mn-doped CsPbCl_3_ was calculated, as shown in Fig. 5(b). Due to the introduction of Mn as a dopant in CsPbCl_3_, different states were introduced in the energy gap closer to the VBO with an energy of −1.231 eV. The CBO moves downward with an energy 0.850 eV compared to the undoped CsPbCl_3_. This leads to a d-band to d-band transition due to the incorporation of the Mn^2+^ ion in CsPbCl_3_. The energy gap between the two d-bands was found to be 2.08 eV. Both the VBO and CBO for Mn-doped CsPbCl_3_ lie at the *Γ*, point which indicates their direct band gap nature with an energy gap of 3.12 eV. These energy gaps in Mn-doped CsPbCl_3_ are responsible for the two observed emission peaks, which are caused by the band offset emission in the CsPbCl_3_ host NCs together with the d-band to d-band transition of the Mn^2+^ ions. These energy gap values are in accordance with the experimentally calculated values from the optical properties. To examine the effect of the Mn dopant in CsPbCl_3_ on the type of semiconductor, we calculated the position of the chemical potential of CsPbCl_3_ and Mn-doped CsPbCl_3_ using DFT based *ab initio* calculations. The evaluated chemical potential values for CsPbCl_3_ and Mn-doped CsPbCl_3_ are −3.871 eV and −3.508 eV, respectively. These values clearly indicate a shift in the chemical potential towards the conduction band due to the incorporation of Mn as a dopant, resulting in n-type behavior in the Mn-doped CsPbCl_3_ NCs.

**Fig. 6:**
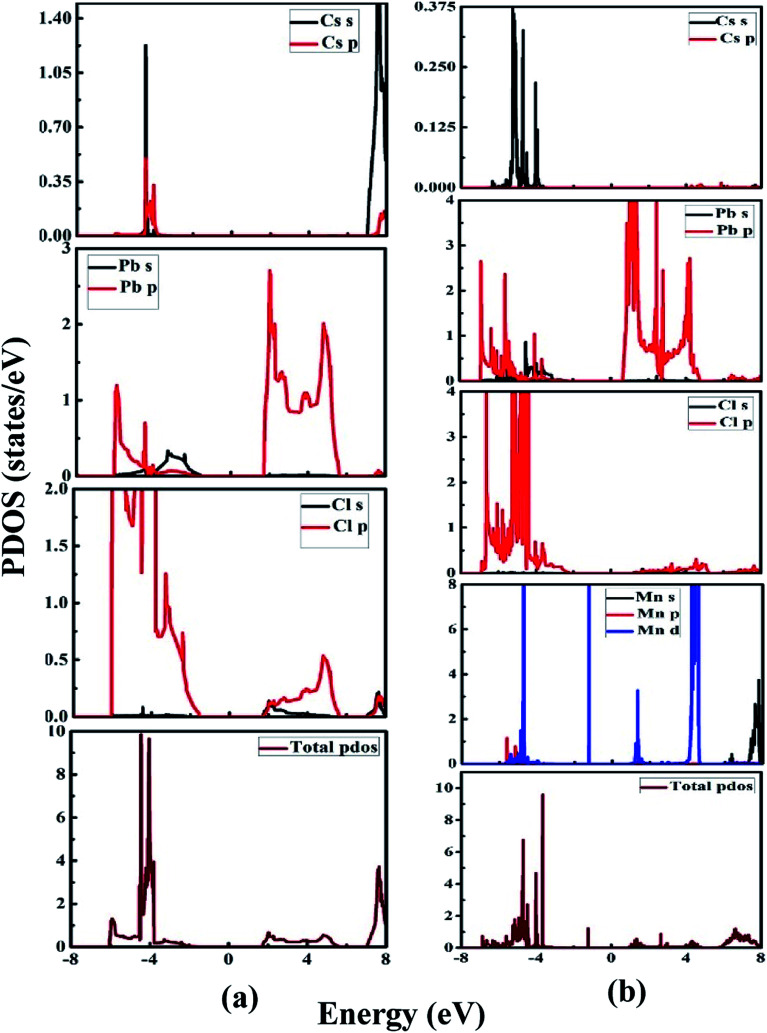
PDOS of (a) CsPbCl_3_ and (b) Mn-doped CsPbCl_3_.

Fig. 6(a) and (b) illustrate the partial density of states of the undoped CsPbCl_3_ and Mn-doped CsPbCl_3_, respectively. By looking at the PDOS of CsPbCl_3_, as shown in Fig. 6(a), the conduction band is mainly due to the electrons of the Pb (6p) orbitals, while the upper valence band is mainly due to the Cl (3p) orbitals. These contributions lead to the emission at band-edge in the CsPbCl_3_ NCs. Cs does not have any significant contribution to conduction band and valence band.

However, Fig. 6(b) shows the PDOS of Mn-doped CsPbCl_3_ NCs, which clearly illustrates the contribution of the Mn (3d) orbitals to the upper valence band and conduction band together with the contribution of Pb (6p) and Cl (3p) orbitals. Moreover, due to the introduction of states by the 3d-orbitals of the Mn^2+^ ions, the energy difference between the d-band to d-band is less than the energy gap of the host CsPbCl_3_ NCs, which can be also seen in the band structure plot in Fig. 5(b). These calculated PDOS results indicate the dual color emission, one due to the host CsPbCl_3_ NCs and the other due to the d-band to d-band transition in Mn^2+^ ions for the Mn-doped CsPbCl_3_.

**Fig. 7:**
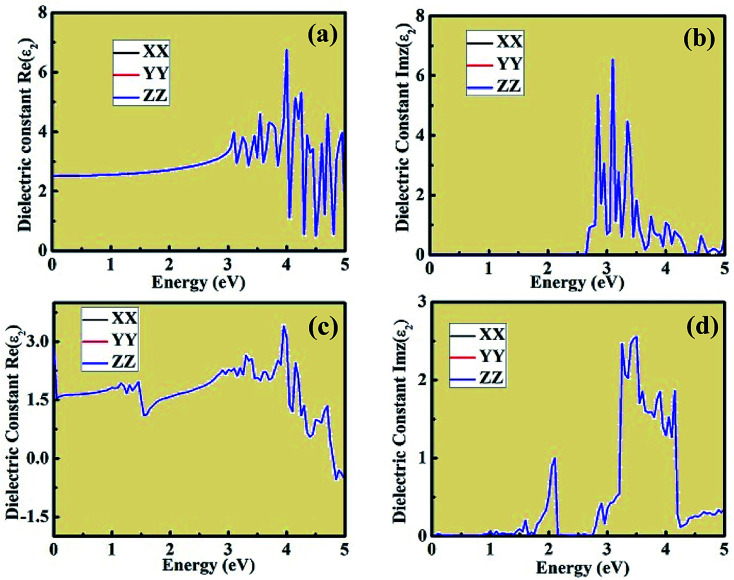
Real and imaginary components of the dielectric constant plot with respect to energy for (a) and (b) CsPbCl_3_, and (c) and (d) Mn-doped CsPbCl_3_ across the *XX*, *YY*, and *ZZ* tensors, respectively.

The dielectric constant plots as a function of energy explain the interaction between the incident light energy and the crystal structure. The real component of the dielectric constant gives information about the anomalous dispersion effects and polarization. However, the imaginary plot of dielectric constant provides a description about the major absorption energy in a crystal structure as a result of neutral charge excitations. These neutral charge excitations lead to a variation in charge density on account of the creation of excitons. To study these aspects, we investigated the optical properties of CsPbCl_3_ and Mn-doped CsPbCl_3_ using *ab initio* DFT calculations. Fig. 7 shows the real and imaginary parts of the evaluated dielectric constant as a measure of photon energy across the three tensors *XX*, *YY*, *ZZ* for both CsPbCl_3_ and Mn-doped CsPbCl_3_. The imaginary component of dielectric constant is an important aspect to examine the optical absorption of crystal structures, which explains the plot of Imz(*ε*_2_) across the *XX*, *YY*, and *ZZ* directions. For the case of CsPbCl_3_, the first significant peak lies at around 2.97 eV, while the strongest peak lies at around 3.12 eV. For the case of Mn-doped CsPbCl_3_, the first significant peak lies at around 2.08 eV, which is attributed to the Mn^2+^ ions (d-band to d-band transition), while the significant peak due to the host CsPbCl_3_ NCs lies at around 3.11 eV. These peaks are in good agreement with the experimentally observed energy gap from the optical properties for undoped CsPbCl_3_ and Mn-doped CsPbCl_3_. The values of the static dielectric constant for CsPbCl_3_ and Mn-doped CsPbCl_3_ are 2.513, and 2.899, respectively, which were evaluated from the real part of the dielectric constant plots. These peaks in the dielectric constant plots indicate that the binding energy of the excitons becomes smaller due to their energy range, resulting in high optical absorption. The effect of Mn doping in CsPbCl_3_ can be seen through the blue-shift phenomenon in the imaginary part of the dielectric constant spectrum as a function of energy.

**Fig. 8:**
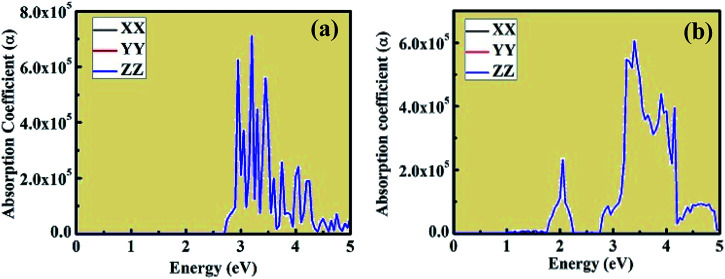
Optical absorption coefficient of CsPbCl_3_ and Mn-doped CsPbCl_3_.

The optical absorption coefficient plots are shown in Fig. 8(a) and (b). The shape of the optical absorption plots shows similar characteristics compared to the imaginary value of dielectric constant plots for both crystal structures. The strongest peak in the optical absorption spectrum lies in the visible region for both CsPbCl_3_ and Mn-doped CsPbCl_3_. In the case of Mn-doped CsPbCl_3_, the optical absorption coefficient plot shows another significant peak at 2.08 eV, which is due to the d-band to d-band transition caused by the Mn^2+^ ions. The absorption coefficient edge lies at 2.97 eV and 3.11 eV for CsPbCl_3_ and Mn-doped CsPbCl_3_, respectively, which correspond to their band gap.

**Fig. 9:**
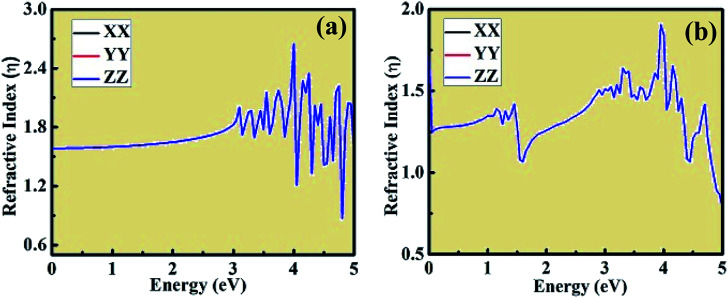
Refractive index plot of (a) CsPbCl_3_ and (b) Mn-doped CsPbCl_3_.

Using the dielectric constant plot, the refractive index across the *XX*, *YY* and *ZZ* tensors were evaluated for both crystal structures. The peaks observed in Fig. 9 follow the trend of the real component of dielectric constant, as shown in Fig. 7(a) and (c). The refractive index plots are also consistent with the dielectric constant and optical absorption plots. Moreover, from the plot of the above calculated optical properties, it was observed that the *XX*, *YY*, and *ZZ* tensors coincide with each other, showing the isotropic behavior of both crystal structures. This type of *ab initio*-based DFT investigation combined with experiment opens a new way to gain insight into the properties of these materials and also about the types of doping needed to tailor their unique and interesting behavior for different applications in the domain of optoelectronics, optomagnetics, and photovoltaics.

## 5. Conclusion

Herein, we presented a theoretical investigation combined with experimental analysis of the structural, electronic and optical properties of inorganic wide energy gap CsPbCl_3_ and Mn-doped CsPbCl_3_ compounds. By introducing Mn as an impurity dopant in CsPbCl_3_, a blue-shift phenomenon was observed, which indicates an increase in the energy gap. A dual color emission was observed in the Mn-doped CsPbCl_3_ compound. Also, the change in chemical potential suggests n-type behavior in Mn-doped CsPbCl_3_. The incorporation of Mn in CsPbCl_3_ led to the introduction of states, which is due to the Mn (3d) orbitals. This behavior can also be easily seen in the plots of the imaginary dielectric constant and absorption coefficient in the form of a significant peak at around 2.08 eV. These theoretical results show very good agreement with our experimentally observed results. Overall, this work presents insights into CsPbCl_3_ and the effect of Mn dopant on its structural, electronic and optical properties, and also suggests a strategy for the synthesis of new compounds with required interesting properties based on various optoelectronics, optomagnetics and photovoltaics devices in halide-based inorganic perovskite compounds.

## Conflicts of interest

There are no conflicts to declare.

## Supplementary Material
